# Use of Anti-Retroviral Therapy in Tuberculosis Patients on Second-Line Anti-TB Regimens: A Systematic Review

**DOI:** 10.1371/journal.pone.0047370

**Published:** 2012-11-05

**Authors:** Matthew Arentz, Patricia Pavlinac, Michael E. Kimerling, David J. Horne, Dennis Falzon, Holger J. Schünemann, Sarah Royce, Keertan Dheda, Judd L. Walson, The ART study group

**Affiliations:** 1 The University of Washington, Seattle, Washington, United States of America; 2 The Bill and Melinda Gates Foundation, Seattle, Washington, United States of America; 3 World Health Organization, Geneva, Switzerland; 4 McMaster University, Hamilton, Canada; 5 University of California San Francisco, San Francisco, California, United States of America; 6 Institute of Infectious Diseases and Molecular Medicine, The University of Cape Town, Cape Town, South Africa; 7 The Division of Pulmonology, The University of Cape Town, Cape Town, South Africa; 8 UCT Lung Institute, The University of Cape Town, Cape Town, South Africa; University of New South Wales, Australia

## Abstract

**Introduction:**

Use of antiretroviral therapy (ART) during treatment of drug susceptible tuberculosis (TB) improves survival. However, data from HIV infected individuals with drug resistant TB are lacking. Second line TB drugs when combined with ART may increase drug interactions and lead to higher rates of toxicity and greater noncompliance. This systematic review sought to determine the benefit of ART in the setting of second line drug therapy for drug resistant TB.

**Methods:**

We included individual patient data from studies that evaluated treatment of drug-resistant tuberculosis in HIV-1 infected individuals published between January 1980 and December of 2009. We evaluated the effect of ART on treatment outcomes, time to smear and culture conversion, and adverse events.

**Results:**

Ten observational studies, including data from 217 subjects, were analyzed. Patients using ART during TB treatment had increased likelihood of cure (hazard ratio (HR) 3.4, 95% CI 1.6–7.4) and decreased likelihood of death (HR 0.4, 95% CI 0.3–0.6) during treatment for drug resistant TB. These associations remained significant in patients with a CD4 less than 200 cells/mm^3^ and less than 50 cells/mm^3^, and when correcting for drug resistance pattern.

**Limitations:**

We identified only observational studies from which individual patient data could be drawn. Limitations in study design, and heterogeneity in a number of the outcomes of interest had the potential to introduce bias.

**Discussion:**

While there are insufficient data to determine if ART use increases adverse drug interactions when used with second line TB drugs, ART use during treatment of drug resistant TB appears to improve cure rates and decrease risk of death. All individuals with HIV appear to benefit from ART use during treatment for TB.

## Introductio

Drug resistant tuberculosis (DR-TB) poses a threat to global health, particularly in regions most affected by the human immunodeficiency virus (HIV) pandemic [1]. A large burden of DR-TB cases occur in Africa, where two-thirds of all HIV infected individuals reside [1]. However, limited access to mycobacterial culture and drug susceptibility testing in settings where HIV/AIDS is most prevalent precludes accurate estimates of DR-TB in these regions [1].

International guidelines recommend that antiretroviral therapy (ART) be started as soon as possible after TB treatment is initiated in patients with HIV and TB [2]–[6]. However, it is not clear if the benefit of early ART extends to individuals on second-line TB treatment regimens for DR-TB. Individuals on second line TB drugs, particularly those with HIV, may experience more side effects, more overlapping toxicities with ART, and have higher rates of non-adherence with TB therapy [7]. Given that second-line treatment may be associated with higher rates of adverse treatment outcomes and higher default rates, evidence based strategies are needed for the management of HIV infected individuals with DR-TB [2], [8].

We performed a systematic review of the published literature on DR-TB in HIV infected individuals and pooled individual patient data (IPD) from included studies. Potential factors affecting survival, cure, default, adverse events, and treatment failure in this population were evaluated.

## Methods

### Ethics Statement

Prior to data collection, a certification of exemption was approved by the University of Washington Institutional Review Board (IRB). In addition, authors from included studies confirmed that they received IRB approval from their primary institutional affiliation.

### Search and Selection of studies

These data were presented in October of 2010 to the WHO guidelines development group following an invitation to contribute to the 2011 update of the guidelines for programmatic management of drug resistant tuberculosis as an evidence review team [9], [10].

We searched Medline, The Cochrane Register of Controlled Trials, GATEWAY and Embase for articles and conference abstracts published from January 1980 through December of 2009 as described previously [11]. We included studies that utilized an appropriate study design (randomized control trials (RCT), quasi-randomized controlled trials, and cohorts with a concurrent (non-historical) comparison group), and met the following criteria: 1) included HIV-1 infected individuals, 2) documented the use or non-use of ART, 3) documented TB disease by a positive sputum culture, 4) documented resistance to at least one first line drug (rifampin, isoniazid, pyrazinamide, ethambutol), 5) documented the use of at least one anti-tuberculosis medication other than rifampin, isoniazid, pyrazinamide, ethambutol or streptomycin, and 6) collected at least one of our outcomes of interest (all-cause mortality, cure, treatment failure, default, time to smear and/or culture negativity or adverse event). Studies performed in both clinics and hospitals, and published in any language or geographic location, were included. We pre-specified that should data from the published study population be insufficient, individual patient data (IPD) would be considered for inclusion. A representative search strategy is shown in Appendix S1.

MA and PP independently evaluated the titles, abstracts, and descriptor terms of all references identified in the initial search, along with the reference lists of relevant reviews and articles, to determine eligibility. When reviewers disagreed on eligibility, studies were reviewed together and consensus was reached. If an abstract was not available, the abstract was not in English, or the discrepant decision could not be resolved based on the abstract alone, the full text was evaluated or the author contacted to assess eligibility.

The full text articles of all references that passed the abstract-review stage were independently evaluated by MA and PP using a pre-determined screening form. Data were extracted from all full text articles by PP and MA to determine eligibility for inclusion. Studies were reviewed for relevance based on study design, participant characteristics, exposures and outcome measures. Risk of bias was assessed at the outcome level after included data sets were identified.

Because no study presented risk estimates stratified by the pre-determined eligible participants, a decision was made to combine individual patient data from all studies and to evaluate these data following the Cochrane Library Guidelines on the use of individual patient data. Eligible authors were asked to provide baseline characteristics (age, gender), details of TB and ART regimens including length of treatments and regimens, details of other drug therapies (such as cotrimoxazole), and outcomes for DR-TB/HIV co-infected study participants who had an end of treatment outcome and whose ART status was known. In addition, study authors were queried on whether TB treatments were modified based on drug susceptibility patterns, and if so, if information on specific second line TB drug regimens was collected. If the authors were able to provide at least details on the second line TB regimen, whether or not the patient was on ART, and one or more outcomes, the study was included. This systematic review was performed in accordance with the guidelines of the preferred reporting items for systematic reviews and meta-analysis (PRISMA) [12].

### Data extraction and definitions

The following characteristics were extracted from each included study: author, publication status, year of implementation, study design, study type, duration, completeness of follow up, country and location of study, settings, method of recruitment, and number of participants. We classified TB drugs according to the classification used in the WHO guidelines (Groups 1,2,3,4 and 5) [13]. Rifampin, isoniazid, pyrazinamide, ethambutol and streptomycin were defined as first line drugs. All other TB drugs were classified as second line drugs. ART was defined as drugs from any of the following classes: nucleoside (and nucleotide) reverse transcriptase inhibitors (NRTI), non-nucleoside reverse transcriptase inhibitors (NNRTI), protease inhibitors (PI), and integrase inhibitors. Multidrug resistant tuberculosis (MDR-TB) was defined as resistance to isoniazid and rifampin. Extensively drug resistant tuberculosis (XDR-TB) was defined as MDR-TB tuberculosis with resistance to a fluoroquinolone and at least one second line injectable agent. Other drug resistance (ODR-TB) was defined as TB drug resistance requiring a second line drug, but not meeting the definition of MDR-TB. Death was defined as all cause mortality during TB treatment. We accepted each individual study definition of cure, default, adverse event, treatment success, and treatment failure. Smear or culture conversion was defined as the occurrence of smear or culture conversion during therapy, without subsequent positive microbiologic specimens during treatment. Time to smear and/or culture conversion was defined as time to the first of three consecutive negative smears/cultures.

### Data Synthesis and Analysis

Individual patient data from all studies were treated as a single cohort. Time to cure, death, treatment failure and default was defined as length of treatment on second line drugs. The incidence of each outcome reported for each intervention group and compared using hazard ratios and 95% confidence interval (CI) from Cox-proportional Hazards models. Time to event analysis was used to account for between-study differences in opportunity for an event to occur because included studies had variable lengths of treatment and follow-up.

Incidence rates and hazard ratios for adverse events were not calculated, as data on timing of adverse events were not available. We instead compared the occurrence of any adverse event across treatment groups by calculating odds ratios using a two-sided Fisher's exact test. To account for the disproportionate length of follow up time between studies, we stratified analyses by follow-up times of less than 1 year, 1–2 years, and greater than 2 years. Specific types of adverse events were not recorded for most studies therefore could not be assessed individually. All statistical analyses were conducted in STATA 10.1 with statistical significance criteria set at p≤0.05.

The quality of evidence was assessed using the GRADE approach [14]. For purposes of systematic reviews, the GRADE approach defines the quality of a body of evidence as the extent to which one can be confident that an estimate of effect or association is close to the quantity of specific interest. Quality of a body of evidence involves consideration of within-study risk of bias (methodological quality), directness of evidence, heterogeneity, precision of effect estimates and risk of publication bias. Heterogeneity and publication bias were only addressed in studies that had both ART users and non-users and in whom the sample size was greater than 10. The quality rating across studies has four levels: high, moderate, low or very low. The GRADE Profiler software was used for performing the GRADE analyses.

## Results

**Figure 1:**
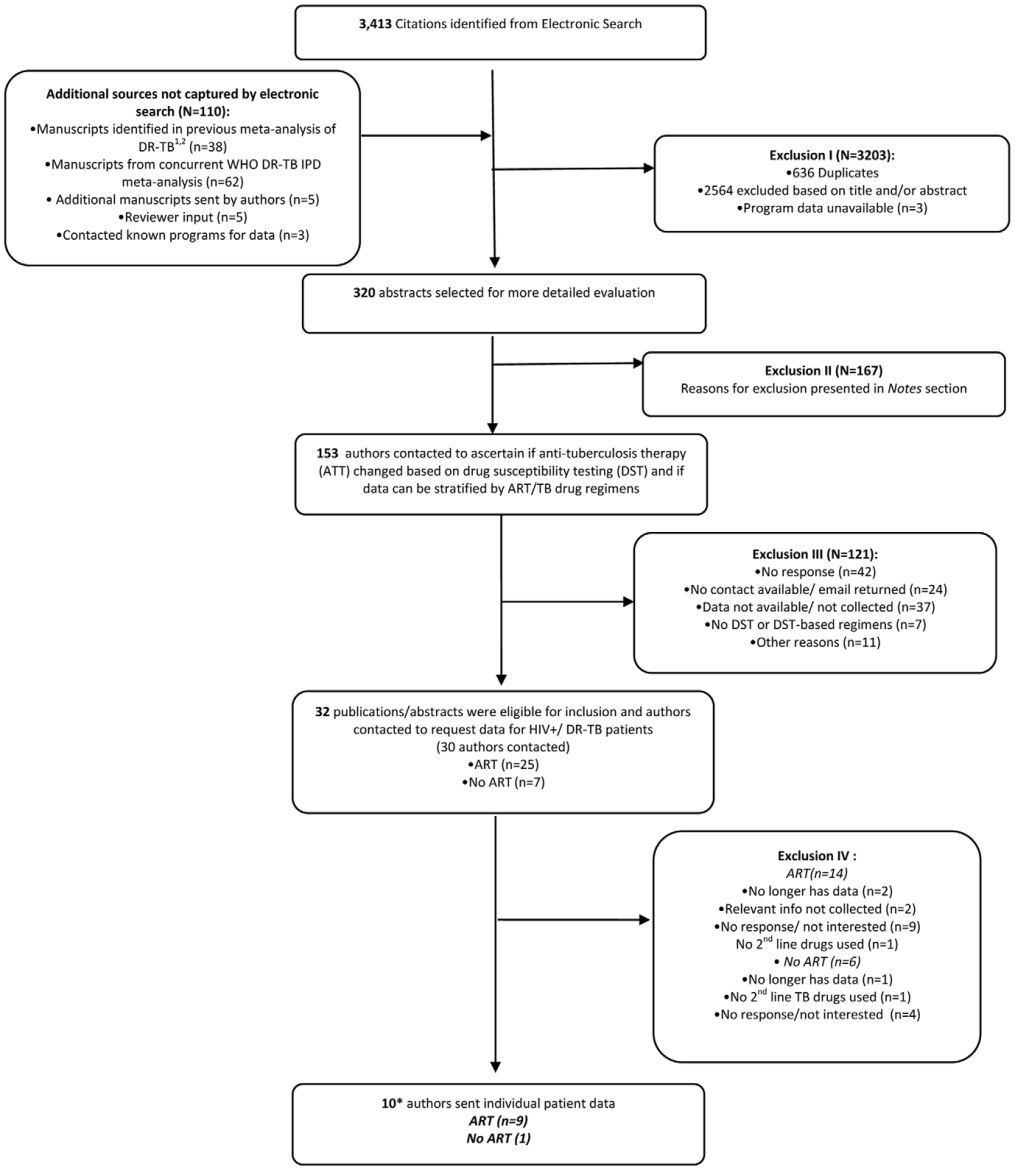
Flow diagram for study inclusion. Two authors each sent data that was represented by two included studies, therefore 12 references actually included. ^1^ Lew W, Pai M, Oxlade O, Martin D and Menzies D. Initial drug resistance and tuberculosis treatment outcomes: systematic review and meta-analysis. Ann Intern Med 2008;149:123–34. ^2^ Menzies D, Benedetti A, Paydar A, et al. Standardized treatment of active tuberculosis in patients with previous treatment and/or with mono-resistance to isoniazid: a systematic review and meta-analysis. PLoS Med 2009; 6:e1000150. **Reasons for Exclusion II.** Inappropriate study design (n = 34); Outcomes of interest are not measured (n = 22); Not deemed research/no data collected (n = 18); No TB drug resistance or drug resistance testing (n = 21); No HIV+ patients or HIV-testing (n = 37); No TB-infected patients or TB treatment (n = 4); No 2^nd^ line drug TB therapy used (n = 16); No ART data collected (n = 3); Author contacted for another study/same patients (n = 12). **Other Reasons for Exclusion III (other n = 11):** Not interested (n = 1); Inappropriate study design (n = 1); No HIV (n = 3); In process of publication (n = 1); Already contacted (n = 5).

Our electronic search strategy resulted in 2777 unique citations. We identified 110 additional citations through review of a previous meta-analysis of studies evaluating treatment outcomes of drug resistant TB and from contact with study authors [15]. We identified 153 potentially relevant studies and contacted the authors of these studies for further information. Eighty-seven authors (57%) responded to our query and 10 individual patient data sets from 12 citations were received for inclusion in our study (figure 1). While all patients from one study met criteria for inclusion in this review [16], only a subset of patients from all other studies met criteria for inclusion. The number patients included from each study and study details are described in table 1.

**Table 1:** Details of included studies.

Study	Methods	Participants	Outcomes Measured in Main study	Definitions
**Varma, 2009**	Prospective cohort study of HIV/TB co-infected patients in Thailand who were programmatically treated and followed from May 2005- September 2006.	Of the 667 patients in the published study, 16 (2.3%) had MDR TB and 273 (41.0%) were prescribed ART. Of published patients, 8 (1.2%) met inclusion criteria, 4 (50%) were on ART. Six (75%) were classified as MDR and 2 (25%) as ODR.	Cure, treatment completion, death, treatment failure, adverse events,	Cure-Not defined in publicationConversion-N/A
**Eker, 2008**	Retrospective hospital-based cohort study of 184 culture confirmed MDR TB patients hospitalized between Jan-2004 through Dec- 2006 in Germany.	Of the 187 patients, 177 (94.7%) were classified as MDR and 7 (3.7%) as XDR. Seven (3.7%) were HIV positive. Four (2.1%) patients were included in the review, all of which were on ART and were classified as MDR.	Cure, treatment completion, treatment success, death, failure, treatment failure (death or failure), default, adverse events, transfer out, culture conversion	Cure-Laserson criteria: completed treatment according to country protocol and consistently negative (≥5 results) in final 12 months of therapy. Conversion-not defined in publication
**Shean, 2008**	Hospital-based retrospective cohort study in South Africa from January 1992- December 2002.	All 491 patients included in the study were MDR and of those tested, 15 (9%) were HIV-infected. Twenty (4.1%) patients were included in the review, all of whom were on ART and all of whom were classified as MDR.	Cure, death, default, transfer out, treatment completion, Culture conversion, smear conversion	Cure-Laserson criteriaConversion-two consecutive negative sputum cultures take a month apart
**Palmero, 2006**	Hospital-based cohort study in Argentina from December 2001 to December 2003.	Of the 53 patients included in the published study, all (100%) were included in the review as well as 1 additional patient. Twenty-eight (51.9%) were on ART. All patients were classified as MDR.	Cure, death, default, adverse event, transfer out	Cure-Laserson criteria. Conversion-N/A
**Migliori, 2007**	Population-based cohort study in Estonia, Germany, Italy, and the Russian Federation between January 1999 and January 2006	Of the 361 MDR and 64 XDR patients included in the published study, 8 (1.9%) patients were included in the review, all were MDR, and all were on ART.	Cure, treatment success, death, default, failure, transfer out, treatment completion, culture conversion, smear conversion	Cure-Laserson criteria. Conversion-not defined in publication
**Jamal, 2003**	Population-based cohort study in Brazil between Jan 1994 and July 2003.^1^	OF the 93 patients sent for possible inclusion, 16 (17.2%) were included in the review, 5 (31.3%) with ODR and 11 (68.8%) classified as MDR. All included patients were on ART.	Cure, transfer out, adverse event	Cure-Negative culture at end of treatment. Conversion-N/A
**Leimane 2010**	Population-based cohort study in Latvia between January 2000 to December 2004.	Of the 1027 patients (979 MDR and 48 XDR) included in the published study, 7 (0.7%) MDR patients were included in the review, 5 (71.4) of which were on ART.	Cure, death, default	Cure-Laserson criteria. Conversion-N/A
**El-Sahly, 2006**	Population-based case-control study^2^ in the US (Houston, Texas) of enrolled TB cases between August 1995 and September 2001.	Of the 193 patients with drug resistance TB in the published study, 10 (0.5%) were classified as having MDR with remaining 183 (94.8%) patients classified as ODR. Nine (4.7%) patients were included in the review, all classified as ODR and all on ART.	Cure, culture conversion, and smear conversion^3^ death,	Cure-Laserson criteria. Conversion-not defined in publication
**Dheda, 2010**	Retrospective cohort study of patients diagnosed and treated at four of nine South African hospitals designated to treat XDR TB in South Africa between August 2002 and February 2008.	Of the 174 XDR patients included in the analysis, 82 (47.1%) were HIV positive and included in the review. Of the included patients, 52 (63%) were on ART.	Death, culture conversion	Cure-N/A. Conversion- Two consecutively negative cultures, collected 1 month apart, with first culture positive at start of treatment.
**Burgos, 2005**	Population-based cohort study in the US (San Francisco) from Jan 1982 to December 2000.	Forty-eight cases of MDR cases were reported, 11 (22.9%) were HIV positive. All HIV positive cases were included in the review, and 2(18.1%) were on ART.	Treatment response, relapse, adverse events, death, culture conversion, smear conversion	Cure- complete course of treatment with microbiologic and clinical response. Conversion-series of 3 negative culture/smear results

1 Data from routine TB surveillance system of the TB Division State of Sao Paulo identified through 2003 thereby representing a larger study population than those included in the referenced abstracts.

2 Nested in larger cohort study, which is the design from which we pulled data.

3 Cure, culture and smear conversion data not presented in publication.

All 10 studies included death as recorded outcome [16]–[25]. Nine of the included studies recorded cure as a potential outcome, although the definition of cure was variably defined [16], [17], [19]–[25]. Three studies included patients with ODR-TB [20], [21], [25]. Of the 16 patients with ODR, 10 had resistance to rifampin, 5 were resistant to isoniazid in addition to a second first line drug other than rifampin, and 1 had isoniazid monoresistance. Six of the eight studies which included patients with MDR-TB defined cure according to the recommended criteria (five or more negative cultures in the final year of treatment) [16], [19], [20], [22]–[24], [26] while other studies defined cure as having two negative cultures in the final month of therapy and a documented clinical response. In the single study including individuals with XDR-TB, cure was not documented [18]. Data on smear or culture conversion were available from six studies [17]–[20], [23], [24]. Default was documented in nine studies and was defined differently across studies. Adverse events were recorded in six studies, although the type of adverse event, the organ system involved, or the severity of the event was not recorded in most studies [16], [17], [19], [21], [24], [25]. Treatment failure was documented in only one patient and was not evaluated as an outcome.

A total of 217 patients were included in our analysis. Included individuals represent a diverse geographic population, with the majority from Africa (47%) and South America (32%). Characteristics of included study participants are shown in table 2. Slightly more than half of all included individuals were male (56%) and most (71%) were between 25 and 44 years of age. Among patients with CD4 count data available at the start of ARV treatment (50% of all included participants), 45% had a CD4 count <50 cells/mm^3^, 85% had CD4 counts <200 cells/mm^3^, 3% had CD4 counts between 200 and 350 cells/mm^3^, and 13% had CD4 counts ≥350 cells/mm^3^. All individuals were being treated for pulmonary TB and more than half (59%) were receiving ART. In the minority of cases (9 patients) the ARV regimen was not known. In the remaining patients, the reasons for choice of ARV regimen were not given and regimens varied widely. No patients were recorded to have received monotherapy for HIV, 4 patients were recorded to have received 2 drug ARV, and the remaining 113 patients received 3 drug ARV. As noted in Table 2, 60 patients received a regimen which included 1 or more non-nucleoside reverse transcriptase inhibitors (NNRTIs), 31 received a regimen with 1 or more nuceloside reverse transcriptase inhibitors (NRTIs), and 26 received a regiment with 1 or more protease inhibitors (PIs). Data on timing of ART initiation relative to TB treatment was available for 17 (8%) patients. While MDR-TB was common (55%), 38% of included individuals were classified as having XDR-TB and 7% with ODR-TB. The median length of TB treatment was 28.5 months (IQR: 10.8–45.8 months). All patients received 4 or more anti-tuberculosis drugs during the intensive phase of treatment.

**Table 2:** Patient characteristics by ART use.

		Antiretroviral Status		
Characteristic	Overall	ART (n = 126) N^1^ (%)	No ART (n = 91) N^1^ (%)	*p*-value^2^
Male				
	86 (55.8)	49 (55.1)	37 (56.9)	0.82
Age (range 18–59)				
<18	1 (0.5)	1(1.0)	0	0.92
18–24	14 (7.6)	7(6.9)	7(8.3)	
25–29	27 (14.6)	16(15.8)	11(13.1)	
30–34	67 (36.2)	34(33.7)	33(39.3)	
35–44	53 (28.7)	31(30.7)	22(26.2)	
45+	23 (12.4)	12(11.9)	11(13.1)	
Median (IQR)	32.9 (29.4–39.5)	33.0 (29.5–39.5)	32.1 (29.3–39.2)	
CD4 count				
>350 cells/µl	13 (12.5)	10(15.2)	3(7.9)	0.550
200–350 cells/µl	3 (2.9)	2(3.0)	1(2.6)	
<200 cells/µl	88 (84.6)	54(81.8)	34(89.5)	
Median (IQR)	55.5 (21.0–123.5)	53.5 (17–155)	56 (23–94)	
TB Resistance Pattern				
ODR	16 (7.4)	15(11.9)	1(1.1)	0.001
MDR	119 (54.8)	59 (46.8)	60(65.9)	
XDR	82 (37.8)	52(41.3)	30(33.0)	
Length of TB treatment				
Median Months (IQR)	28.5 (10.8–45.8)	31.9(15.2–45.8)	22.5 (4.9–43.8)	0.008
Total Number of Drugs				
< = 4	61 (28.1)	39(31.0)	22(24.2)	0.179
5	85 (39.2)	52(41.3)	33(36.3)	
> = 6	71 (32.7)	35(27.8)	36(39.6)	
Total Number of Effective Drugs^3^				
< = 3	185 (85.3)	112 (88.9)	73(80.2)	0.185
4	22 (10.1)	10(7.9)	12(13.2)	
>4	10 (4.6)	4(3.2)	6(6.6)	
ART Regimen Base				
NNRTI	–	60(51.3)	–	–
PI	–	26 (22.2)	–	–
NRTI	–	31 (26.5)	–	–

1 N's may not add up to total N because of missing values.

2 *p*-values of ART use vs. non-use comparisons based on the categorical version of variable are presented unless interpretation of p-value based on of continuous version differed.

3 Effective drug: Demonstrated susceptibility to drug by sputum culture.

**Figure 2:**
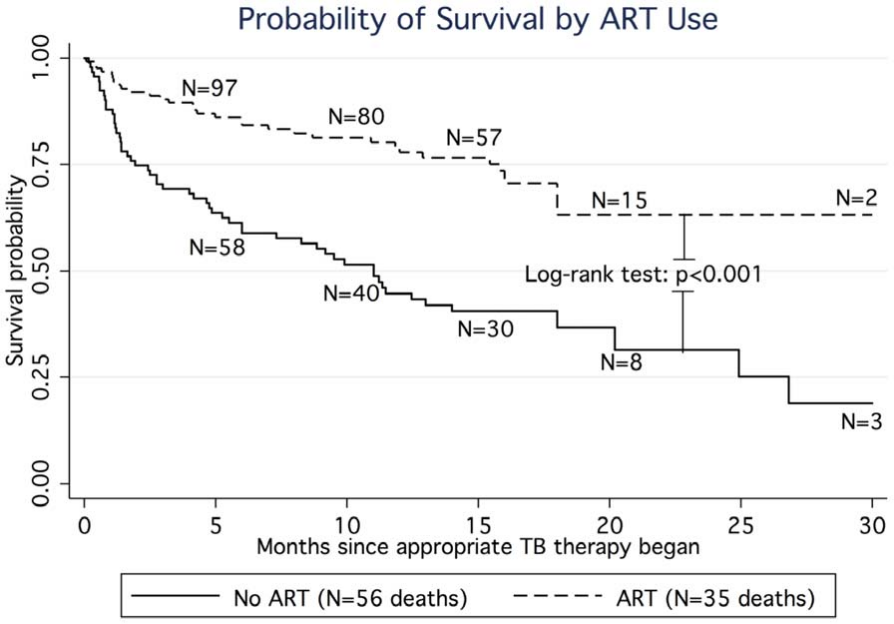
Kaplan Meier Curve for survival comparing ART vs. no ART among all-patients (N = 216). *Note: 1 additional death occurred at month 37*.

Ninety-one patients (42%) died during TB treatment (incidence rate (IR): 434 per 1000 person years (PY)). ART users were less likely to die than ART-non-users (HR: 0.38, 95% CI 0.25, 0.58) (Table 3 & Figure 2) and the median time to death was significantly shorter among ART non-users (11 months) than among ART users (37 months). The association between ART use and survival did not change after adjustment for TB resistance pattern categorized as ODR, MDR, and XDR. The benefit of ART use in reducing risk of death was more pronounced in patients with CD4 counts <200 cells/mm^3^ (HR: 0.24 (95%CI: 0.14, 0.43) and greatest in magnitude among the 47 patients with CD4 counts <50 cells/mm^3^ (HR: 0.15; 95%CI: 0.06, 0.38). A risk estimate describing the association between ART use and death among those patients with CD4 counts ≥350 cells/mm^3^ could not be estimated due to only 1 death occurring among an ART user.

**Table 3:** Association predictors and death, cure, culture conversion, and smear conversion.

	Death		Cure		Culture Conversion		Smear Conversion	
	Unadjusted	Adjusted^a^	Unadjusted	Adjusted^a^	Unadjusted	Adjusted^1^	Unadjusted	Adjusted^2^
	HR (95% CI)	HR (95% CI)	HR (95% CI)	HR (95% CI)	HR (95% CI)	HR (95% CI)	HR (95% CI)	HR (95% CI)
ART use vs. non use	**0.38(0.25, 0.58)**	**0.39 (0.26, 0.61)**	**3.40 (1.56–7.40)**	**2.32 (1.02–5.28)**	1.04 (0.61, 1.80)	1.61 (0.79–3.27)	2.21 (0.97–5.04)	–
CD4 count	**0.56 (0.37, 0.86)**	**0.56 (0.35–0.90)**	1.10 (0.92–1.32)	0.94 (0.75–1.19)	1.00 (0.89–1.11)	1.00 (0.89–1.14)	0.90 (0.63–1.30)	–
Age	1.02 (0.89–1.17)	1.01 (0.89–1.16)	0.94 (0.75–1.19)	0.98 (0.76–1.26)	1.04 (0.88–1.23)	1.07 (0.91, 1.26)	1.03 (0.83–1.29)	–
Female vs. male	1.09 (0.64–1.85)	0.96 (0.56–1.64)	0.75 (0.29–1.94)	0.96 (0.35–2.66)	0.54 (0.29–1.00)	0.67 (0.36–1.28)	0.60 (0.24–1.47)	–

1 Adjusted for TB resistance pattern as indicator variables.

2 Adjustment not possible because less than 6 patients with ODR and none with XDR.

**Figure 3:**
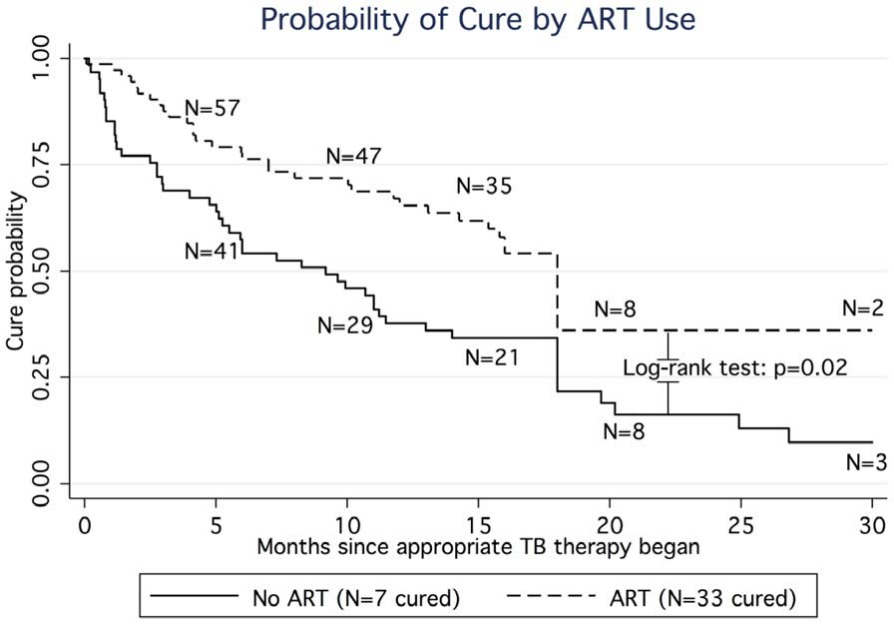
Kaplan Meier Curve for cure comparing ART vs. no ART among all-patients (N = 216). *Note: 1 additional cure event occurred at month 60*.

Approximately one-third of patients (40/134) met the individual study's criteria for cure at the end of treatment (incidence rate of 292 per 1000 PY). Cure was more common in ART users compared to non-users (HR of for cure: 3.4, CI: 1.6, 7.4) (Table 3 & Figure 3). The magnitude of the association between ART use and likelihood of cure was greater among individuals with CD4 counts <200 cells/mm^3^ (HR: 7.44, 95% CI: 1.13, 48.9) as compared to those with CD4 counts ≥350 cells/mm^3^ (HR: 2.66, 95%CI: 0.45, 15.8). However there was not statistical evidence of effect modification by CD4 count (likelihood ratio test p = 0.22). After adjusting for TB resistance pattern, ART use remained associated with a greater likelihood of cure (aHR: 2.3 (1.0, 5.3).

Fifty-two of 121 participants (41%) with culture conversion data available had documented conversion to negative during treatment while 26 (72%) of those with data available converted their smear to negative during treatment. There was no statistically significant association between ART use and earlier time to culture conversion (aHR: 1.86, 95%CI: 0.98, 3.56) or smear conversion (HR: 2.21, 95%CI: 0.97, 5.04).

Default during treatment occurred in 15 (11%) of the 134 participants with data available. Default was not associated with ART use among all included participants, although among individuals with CD4 cell counts <200 cells/mm^3^, ART users did appear less likely to default (HR: 0.26, 95%CI: 0.07, 0.93). Among the 120 individuals with adverse event data, almost a third (28%) had an adverse event recorded: 19 (31%) participants using ART and 15 (25%) of participants not using ART (p = 0.55). Data on severe adverse events were limited, however one of the 19 individuals with an AE while using ART had a documented change in treatment and 4 of the 15 individuals with an AE not using ART had a change in treatment. Among patients followed up for <1 year, 7.7% of ART users had any adverse event whereas 26.3% non-users had an adverse event reported. Conversely, among patients followed for 1 or more years, 48% of ART users had an adverse event reported and 23.8% non-users (p = 0.10).

## Discussion

While large randomized controlled trials of drug susceptible TB using first line anti-tuberculosis drugs have demonstrated survival benefit with the use of ART, similar trials amongst HIV infected individuals with drug resistant tuberculosis are lacking [2]. The results of this analysis suggests that ART increases survival and results in higher rates of TB cure in HIV infected individuals with drug resistant tuberculosis. In addition, ART does not appear to impact frequency of adverse drug reactions or result in higher rates of default from TB treatment programs in these individuals. ART appears to benefit all HIV infected individuals during treatment for tuberculosis.

In this analysis, the benefit of ART among HIV infected individuals with DR-TB was seen across all levels of immunosuppression, although the benefit was most pronounced among those with CD4 counts <50 cells/mm^3^. This finding supports current WHO recommendations to start ART in subjects with HIV and drug resistant tuberculosis irrespective of CD4 count [9].

The benefit in survival and cure observed in this analysis may be due to several mechanisms. First, It is possible that the impact of ART on TB treatment outcomes is due to the survival benefit of ART in HIV infected individuals. ART increases survival through a number of pathways, including a reduction in risk of new opportunistic infections and a reduction in AIDS defining illness [27]–[31]. This effect of ART on survival is strongest among individuals with lower CD4 counts [3], [4]. If HIV infected individuals on ART survive longer, they will have greater opportunity for cure.

It is also likely that the immune reconstitution observed following initiation of ART impacts the control and clearance of TB [32]. The recovery of circulating T cells capable of producing interferon gamma and other Th1 cytokines following the initiation of ART may directly result in improved immune responsiveness, a more robust granulomatous response, and an increase in mycobacterial killing and containment [33].

The increased rates of survival and cure observed may also be the results of the impact of ART on retention into care. Individuals enrolled into ART care are required to make frequent visits to health care facilities and early initiation of ART in patients being treated for TB has been shown to improve retention to completion of TB treatment [34].

Second line treatment options for TB can involve the use of multiple drugs, which may have overlapping potential toxicities with available ART (table 4) [35], [36]. The use of ART in combination with TB drugs also increases pill burden, potentially resulting in lower rates of compliance [37], [38]. In this analysis, we report that the overall risk of adverse events was not higher in individuals who received ART when compared to those who did not receive ART. It is important to note that this analysis had limited power to detect differences in adverse events between individuals stratified by type of ART use. This may be important when considering particular combinations of ARV/SLD, such as tenofovir and group 2 TB drugs, which have overlapping toxicities. In addition, the lack of a common definition of an adverse event across studies and lack of documentation of time to an adverse event further limited this analysis.

**Table 4:** Overlapping toxicities of anti-tuberculosis drugs and ARV.

Potential Toxicity	Antiretroviral Therapy	Antituberculosis Therapy
peripheral neuropathy	stavudine	cycloserine
	didanosine	isoniazid
		ethambutol
		flouroquinolones
		streptomycin
		kanamycin
		amikacin
		capreomycin
		viomycin
		ethionomide/prothionomide
		linezolid
psychiatric symptoms	efavirenz	cycloserine
		isoniazid
		flouroquinolones
		ethionomide/prothionomide
hepatitis	nevirapine	pyrazinamide
	ritonovir/protease inhibitors	isoniazid
	efavirenz	rifampin/rifabutin
	etravirine	para-aminosalicylic acid
	maraviroc	ethionomide/prothionomide
		flouroquinolones
renal toxicity	tenofovir	streptomycin
	indinavir	kanamycin
		capreomycin
		amikacin
		viomycin
		rifampin
gastro-intestinal intolerance	zidovudine	ethionomide/prothionomide
	protease inhibitors	para-aminosalicylic acid
	stavudine	pyrazinamide
	didanosine	isoniazid
		rifampin
		ethambutol
		clofazimine
bone marrow toxicity	zidovudine	linezolid
		rifampin (thrombocytopenia)
lactic acidosis	stavudine	linezolid
	didanosine	
	zidovudine	
stevens-johnson syndrome	nevirapine	thioacetazone
	efavirenz	cycloserine
	etravirine	linezolid
		ethambutol
		streptomycin
arrhythmias/Qt prolongation	atazanavir	flouroquinolones
	saquinavir/ritonavir	
	Kaletra	
rash/pruritus	nevirapine	rifampin/rifabutin
	efavirenz	pyrazinamide
	etravirine	
	abacavir	

We found ART use was associated with a trend toward increased smear conversion and a shorter time to conversion. Given that second line TB regimens may be less effective at clearing TB infection, this finding suggests that ART may provide some additive benefit in reducing bacillary load in these patients [39]. If confirmed, ART may be an important adjunct to appropriate infection control by reducing smear positivity in areas with high rates of drug resistance and TB/HIV co-infection.

Strengths of this systematic review include the relatively large number of individuals included in the pooled analysis. In addition, the analysis was performed according to published guidelines for systematic reviews. However, there are a number of limitations. The inclusion of observational studies may have resulted in selection bias by selecting individuals for ART who were presumed to be more compliant. These individuals would have more frequent contact with health care providers as a result of being on ART, and subsequently more opportunity for diagnosis and management of health issues. ART therefore may be a surrogate for increased access to care and improved compliance with treatment. Ideally, we would perform a comprehensive analysis of statistical heterogeneity across studies. However, meta-regression requires that effect size be estimated within each study. Because some studies contributed a very small number of patients, or only contributed a subset of patients either on or off ART, within-study effect estimates were not possible for all studies. However, when we did an analysis of the subset of studies with sufficient sample size [16], [18] we found homogeneity in survival estimates. We were also unsuccessful in contacting 43% of authors with potential data for this analysis. In addition, we may have failed to identify other sources of data due to publication bias. Significant heterogeneity was observed in the outcomes measured and in the definition of recorded outcomes. This resulted in few individuals being included in the analyses of some outcomes of interest, which limited our power to evaluate many of these outcomes. Lastly, three randomized controlled trials evaluating ART timing and use in TB cases were published after our search strategy was employed [2], [4], [6]. Although these studies enrolled mostly drug sensitive TB patients, the data from drug resistant TB patients could have improved the GRADE quality of the studies. limitations contributed to a very low quality of GRADE evidence for the early initiation of ART in all subjects with drug resistant tuberculosis [10].

Despite these limitations, the results of this analysis suggest a benefit of ART use in patients with drug resistant TB and HIV [9]. This analysis has highlighted important gaps in the literature regarding the optimal management of drug resistant TB in HIV infected patients. Prospective studies comparing different ART regimens in individuals with drug resistant tuberculosis requiring second line anti-tuberculosis drugs are needed. In addition, HIV infected individuals should be included in trials evaluating new anti-tuberculosis drugs and regimens for drug resistant tuberculosis [40]. Given that a substantial proportion of drug resistant TB is emerging among HIV infected individuals, optimal treatment strategies are needed to direct the management of these individuals.

## Supporting Information

Table S1 **Example of the search strategy as employed in Gateway from January of 1980 to December of 2009.**
(DOC)

Table S2 **PRISMA 2009 checklist.**
(DOC)

Appendix S1 (DOC)
