# Supplementary material for: Use of Anti-Retroviral Therapy in Tuberculosis Patients on Second-Line Anti-TB Regimens: A Systematic Review
Source: PLoS One. 2012 Nov 5;7(11):e47370. doi: 10.1371/journal.pone.0047370 (PMC3489892; doi:10.1371/journal.pone.0047370)
Supplement: Table S1 — Example of the search strategy as employed in Gateway from January of 1980 to December of 2009. (DOC) [file pone.0047370.s001.doc]

**Table S1: Search Strategy**

**Title: Treatment of drug-resistant tuberculosis in patients with HIV-1 infection**

# Database: GATEWAY (1980 – 2009)

**Date:** **11 December 2009**

**Number of meeting abstracts retrieved: 280 records**

| Search Number | Search | Items Found |
| --- | --- | --- |
| #3 | Search: ((drug resistance OR resistance AND tuberculosis) OR multidrug-resistant tuberculosis OR extensively drug-resistant tuberculosis OR "mdr tb" OR "xdr tb" OR extremely drug-resistant tuberculosis) AND (((((HIV INFECTIONS) OR HIV OR HIV OR HIV-1* OR HIV-2* OR HIV1 OR HIV2 OR (HIV INFECT*) OR (HUMAN IMMUNODEFICIENCY VIRUS) OR (HUMAN IMMUNEDEFICIENCY VIRUS) OR (HUMAN IMMUNO-DEFICIENCY VIRUS) OR (HUMAN IMMUNE-DEFICIENCY VIRUS) OR ((HUMAN IMMUN*) AND (DEFICIENCY VIRUS)) OR (ACQUIRED IMMUNODEFICIENCY SYNDROME)))) OR ((((ACQUIRED IMMUNEDEFICIENCY SYNDROME) OR (ACQUIRED IMMUNO-DEFICIENCY SYNDROME) OR (ACQUIRED IMMUNE-DEFICIENCY SYNDROME) OR ((ACQUIRED IMMUN*) AND (DEFICIENCY SYNDROME)) OR (SEXUALLY TRANSMITTED DISEASES, VIRAL))))) Limit: 1980/01/01:2009/12/11 | 2430 |
| #2 | Search: ((((HIV INFECTIONS) OR HIV OR HIV OR HIV-1* OR HIV-2* OR HIV1 OR HIV2 OR (HIV INFECT*) OR (HUMAN IMMUNODEFICIENCY VIRUS) OR (HUMAN IMMUNEDEFICIENCY VIRUS) OR (HUMAN IMMUNO-DEFICIENCY VIRUS) OR (HUMAN IMMUNE-DEFICIENCY VIRUS) OR ((HUMAN IMMUN*) AND (DEFICIENCY VIRUS)) OR (ACQUIRED IMMUNODEFICIENCY SYNDROME)))) OR ((((ACQUIRED IMMUNEDEFICIENCY SYNDROME) OR (ACQUIRED IMMUNO-DEFICIENCY SYNDROME) OR (ACQUIRED IMMUNE-DEFICIENCY SYNDROME) OR ((ACQUIRED IMMUN*) AND (DEFICIENCY SYNDROME)) OR (SEXUALLY TRANSMITTED DISEASES, VIRAL)))) | 379518 |
| #1 | Search: (drug resistance OR resistance AND tuberculosis) OR multidrug-resistant tuberculosis OR extensively drug-resistant tuberculosis OR "mdr tb" OR "xdr tb" OR extremely drug-resistant tuberculosis | 13816 |
